# 5-(3-Meth­oxy­phen­yl)-3-phenyl-1,2-oxazole

**DOI:** 10.1107/S160053681300740X

**Published:** 2013-03-28

**Authors:** B. Balakrishnan, C. Praveen, P. R. Seshadri, P. T. Perumal

**Affiliations:** a Department of Physics, P.T. Lee Chengalvaraya Naicker College of Engineering and Technology, Kancheepuram 631 502, India; b Organic Chemistry Division, Central Leather Research Institute, Chennai 600 020, India; c Postgraduate and Research Department of Physics, Agurchand Manmull Jain College, Chennai 600 114, India

## Abstract

In the title compound, C_16_H_13_NO_2_, the isoxazole ring makes dihedral angles of 17.1 (1)° with the 3-meth­oxy­phenyl ring and 15.2 (1)° with the phenyl group. Centrosymmetric dimers that are realised by pairs of C—H⋯π inter­actions are observed in the crystal structure.

## Related literature

For general background to isoxazole derivaties, see: Sperry & Wright (2005 ▶); Tanaka *et al.* (2007 ▶). For their biological activity, see: Stevens & Albizati (1984 ▶). For related structures, see: Samshuddin *et al.* (2011 ▶); Balakrishnan *et al.* (2011 ▶).
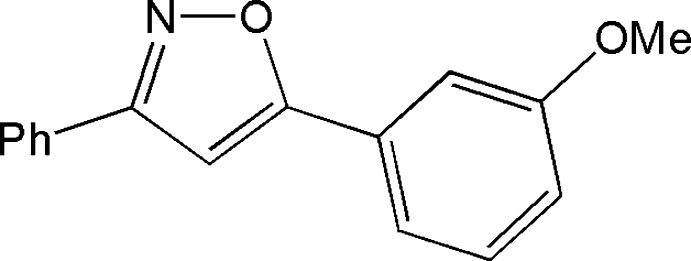


## Experimental

### 

#### Crystal data

C_16_H_13_NO_2_
*M* *_r_* = 251.27
Orthorhombic, 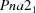

*a* = 7.909 (2) Å
*b* = 27.239 (8) Å
*c* = 5.9652 (17) Å
*V* = 1285.1 (6) Å^3^
*Z* = 4
Mo *K*α radiation
μ = 0.09 mm^−1^
*T* = 295 K
0.35 × 0.30 × 0.30 mm

#### Data collection

Bruker Kappa APEXII CCD diffractometer
6838 measured reflections
2898 independent reflections
2256 reflections with *I* > 2σ(*I*)
*R*_int_ = 0.031

#### Refinement

*R*[*F* ^2^ > 2σ(*F* ^2^)] = 0.038
*wR*(*F* ^2^) = 0.103
*S* = 1.03
2898 reflections
174 parameters
2 restraints
H-atom parameters constrained
Δρ_max_ = 0.13 e Å^−3^
Δρ_min_ = −0.13 e Å^−3^

### 

Data collection: *APEX2* (Bruker, 2008 ▶); cell refinement: *SAINT* (Bruker, 2008 ▶); data reduction: *SAINT*; program(s) used to solve structure: *SHELXS97* (Sheldrick, 2008 ▶); program(s) used to refine structure: *SHELXL97* (Sheldrick, 2008 ▶); molecular graphics: *ORTEP-3 for Windows* (Farrugia, 2012 ▶); software used to prepare material for publication: *SHELXL97*, *PLATON* (Spek, 2009 ▶) and *publCIF* (Westrip, 2010 ▶).

## Supplementary Material

Crystal structure: contains datablock(s) I, global. DOI: 10.1107/S160053681300740X/zj2101sup1.cif

Structure factors: contains datablock(s) I. DOI: 10.1107/S160053681300740X/zj2101Isup2.hkl

Supplementary material file. DOI: 10.1107/S160053681300740X/zj2101Isup3.cml

Additional supplementary materials:  crystallographic information; 3D view; checkCIF report

## Figures and Tables

**Table 1:** Hydrogen-bond geometry (Å, °) *Cg* is the centroid of the C1–C6 ring.

*D*—H⋯*A*	*D*—H	H⋯*A*	*D*⋯*A*	*D*—H⋯*A*
C1—H1⋯*Cg* ^i^	0.93	2.96	3.732 (2)	141
C4—H4⋯*Cg* ^ii^	0.93	3.06	3.768 (3)	134

Symmetry codes: (i) 
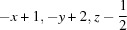
; (ii) 
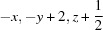
.

**Fig. 1.**
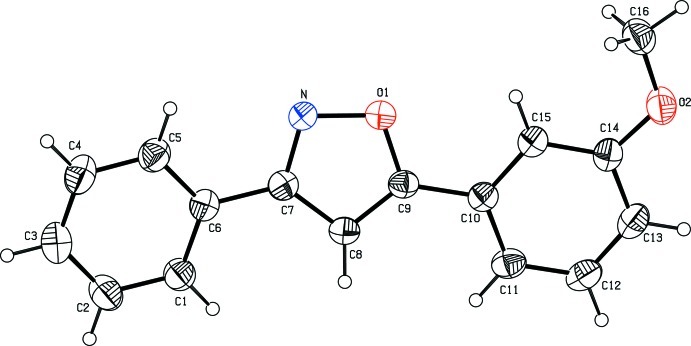
Molecular structure of the title compound, showing 30% probability displacement ellipsoids.

**Fig. 2.**
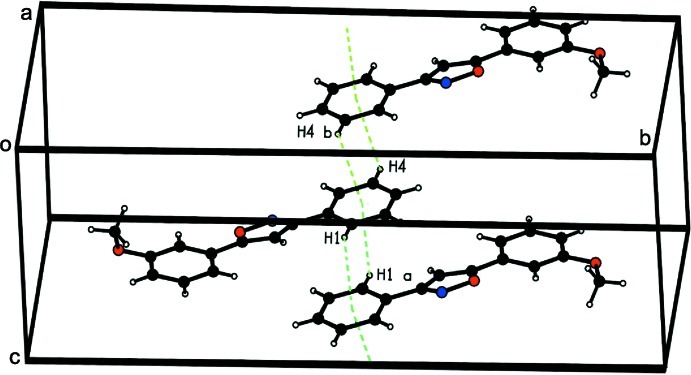
A view of the C—H···π interactions in the crystal structure of the title compound.

## supplementary crystallographic information

### Comment

Isoxazoles are important class of heteroaromatic molecules which are components in a variety of natural products and medicinally useful compounds (Sperry & Wright, 2005). Isoxazole also finds application in organic synthesis as synthetic intermediates and chiral ligands. The liquid crystalline property of isoxazole derivatives and its potential application in optoelectric devices made them attractive synthetic target (Tanaka *et al.*, 2007)

Isoxazole derivaties bearing different substituents are known to have various biological activities in pharmaceutical and agricultural areas. Isoxazole compounds have been widely studied because they exhibit some fungicidal activity, plant -growth regulating activity and antibacterial activity (Stevens & Albizati, 1984). In the title compound the isoxazole ring makes a dihedral angle of 17.1 (1)° with methoxy phenyl ring C10/C11/C12/C13/C14/C15/O2/C16 and a dihedral angle of 15.2 (1)° with the phenyl ring C1/C2/C3/C4/C5/C6 attached to the planar isoxazole moiety.The geometrical parameters agree well with the reported structure (Samshuddin *et al.*2011; Balakrishnan *et al.* 2011). A Centrosymmetric dimers are formed by C—H···π (C1—H1···*Cg* and C5—H5···*Cg*) interactions, where *Cg* is the centroid of the ring C1—C6 (Fig. 2).

### Experimental

To solution of 1-phenyl-3-*m*-tolyl-propynone oxime (251 mg, 1.0 (mmol) in dry dichloromethane (1 ml) was added AuCl_3_ (3.03 mg, 1 mol%) under N_2_ atmosphere and stirred for 10 min. After completion of the reaction as indicated by TLC, the reaction mixture was concentrated under reduced pressure and purified by colomn chromatography over silica gel (100–200 mech) using EtOAc/hexane to afford the pure product.

### Refinement

All H atoms were positioned geometrically and allowed to ride on their parent atoms, with C—H=0.93–0.97 Å and *U*_iso_ (H)= 1.5Ueq(C) for methyl H atoms and 1.2 *U*_eq_(C) for other H atoms.

### Crystal data

**Table:**

C_16_H_13_NO_2_	*F*(000) = 528
*M**_r_* = 251.27	*D*_x_ = 1.299 Mg m^−^^3^
Orthorhombic, *P**n**a*2_1_	Mo *K*α radiation, λ = 0.71073 Å
Hall symbol: P 2c -2n	Cell parameters from 2593 reflections
*a* = 7.909 (2) Å	θ = 2.7–28.4°
*b* = 27.239 (8) Å	µ = 0.09 mm^−^^1^
*c* = 5.9652 (17) Å	*T* = 295 K
*V* = 1285.1 (6) Å^3^	Block, colourless
*Z* = 4	0.35 × 0.30 × 0.30 mm

### Data collection

**Table:**

Bruker Kappa APEXII CCD diffractometer	2256 reflections with *I* > 2σ(*I*)
Radiation source: fine-focus sealed tube	*R*_int_ = 0.031
Graphite monochromator	θ_max_ = 28.4°, θ_min_ = 2.7°
ω and φ scan	*h* = −10→8
6838 measured reflections	*k* = −36→32
2898 independent reflections	*l* = −7→6

### Refinement

**Table:**

Refinement on *F*^2^	Secondary atom site location: difference Fourier map
Least-squares matrix: full	Hydrogen site location: inferred from neighbouring sites
*R*[*F*^2^ > 2σ(*F*^2^)] = 0.038	H-atom parameters constrained
*wR*(*F*^2^) = 0.103	*w* = 1/[σ^2^(*F*_o_^2^) + (0.052*P*)^2^ + 0.0082*P*] where *P* = (*F*_o_^2^ + 2*F*_c_^2^)/3
*S* = 1.03	(Δ/σ)_max_ < 0.001
2898 reflections	Δρ_max_ = 0.13 e Å^−^^3^
174 parameters	Δρ_min_ = −0.13 e Å^−^^3^
2 restraints	Extinction correction: *SHELXL97* (Sheldrick, 2008), Fc^*^=kFc[1+0.001xFc^2^λ^3^/sin(2θ)]^-1/4^
Primary atom site location: structure-invariant direct methods	Extinction coefficient: 0.0071 (19)

### Special details

**Table:**

Geometry. All e.s.d.'s (except the e.s.d. in the dihedral angle between two l.s. planes) are estimated using the full covariance matrix. The cell e.s.d.'s are taken into account individually in the estimation of e.s.d.'s in distances, angles and torsion angles; correlations between e.s.d.'s in cell parameters are only used when they are defined by crystal symmetry. An approximate (isotropic) treatment of cell e.s.d.'s is used for estimating e.s.d.'s involving l.s. planes.
Refinement. Refinement of *F*^2^ against ALL reflections. The weighted *R*-factor *wR* and goodness of fit *S* are based on *F*^2^, conventional *R*-factors *R* are based on *F*, with *F* set to zero for negative *F*^2^. The threshold expression of *F*^2^ > σ(*F*^2^) is used only for calculating *R*-factors(gt) *etc*. and is not relevant to the choice of reflections for refinement. *R*-factors based on *F*^2^ are statistically about twice as large as those based on *F*, and *R*- factors based on ALL data will be even larger.

### Fractional atomic coordinates and isotropic or equivalent isotropic displacement parameters (Å^2^)

**Table:**

	*x*	*y*	*z*	*U*_iso_*/*U*_eq_	
C1	0.6539 (2)	0.00884 (6)	0.6796 (4)	0.0597 (5)	
H1	0.5895	0.0187	0.8020	0.072*	
C2	0.7022 (3)	−0.03964 (7)	0.6594 (4)	0.0713 (6)	
H2	0.6680	−0.0624	0.7665	0.086*	
C3	0.8006 (3)	−0.05449 (7)	0.4817 (4)	0.0735 (6)	
H3	0.8334	−0.0872	0.4693	0.088*	
C4	0.8501 (3)	−0.02114 (8)	0.3231 (5)	0.0739 (6)	
H4	0.9178	−0.0311	0.2041	0.089*	
C5	0.7994 (2)	0.02776 (7)	0.3393 (4)	0.0640 (5)	
H5	0.8317	0.0502	0.2299	0.077*	
C6	0.70095 (19)	0.04300 (6)	0.5184 (3)	0.0494 (4)	
C7	0.6472 (2)	0.09484 (6)	0.5388 (3)	0.0485 (4)	
C8	0.5818 (2)	0.11998 (6)	0.7237 (3)	0.0493 (4)	
H8	0.5592	0.1074	0.8655	0.059*	
C9	0.5583 (2)	0.16697 (6)	0.6515 (3)	0.0471 (4)	
C10	0.49808 (19)	0.21163 (6)	0.7641 (3)	0.0476 (4)	
C11	0.4136 (2)	0.20828 (7)	0.9665 (3)	0.0584 (4)	
H11	0.3926	0.1777	1.0304	0.070*	
C12	0.3606 (2)	0.25068 (8)	1.0733 (3)	0.0637 (5)	
H12	0.3037	0.2485	1.2093	0.076*	
C13	0.3916 (2)	0.29611 (7)	0.9793 (3)	0.0619 (5)	
H13	0.3544	0.3244	1.0514	0.074*	
C14	0.4776 (2)	0.29962 (6)	0.7785 (3)	0.0520 (4)	
C15	0.5295 (2)	0.25759 (6)	0.6679 (3)	0.0503 (4)	
H15	0.5848	0.2599	0.5307	0.060*	
C16	0.5937 (3)	0.35244 (7)	0.4984 (4)	0.0720 (6)	
H16A	0.5336	0.3357	0.3813	0.108*	
H16B	0.7054	0.3390	0.5115	0.108*	
H16C	0.6011	0.3868	0.4631	0.108*	
N	0.6620 (2)	0.12403 (5)	0.3651 (3)	0.0628 (4)	
O1	0.60346 (19)	0.17058 (5)	0.4372 (2)	0.0689 (4)	
O2	0.50660 (17)	0.34640 (5)	0.7034 (2)	0.0704 (4)	

### Atomic displacement parameters (Å^2^)

**Table:**

	*U*^11^	*U*^22^	*U*^33^	*U*^12^	*U*^13^	*U*^23^
C1	0.0586 (10)	0.0583 (10)	0.0624 (12)	0.0025 (8)	0.0068 (10)	0.0017 (9)
C2	0.0738 (12)	0.0572 (11)	0.0830 (16)	0.0021 (9)	0.0046 (12)	0.0100 (10)
C3	0.0691 (12)	0.0575 (11)	0.0939 (18)	0.0050 (9)	−0.0027 (13)	−0.0120 (11)
C4	0.0713 (12)	0.0695 (12)	0.0807 (16)	−0.0010 (10)	0.0139 (12)	−0.0203 (11)
C5	0.0665 (11)	0.0624 (11)	0.0629 (12)	−0.0055 (9)	0.0102 (10)	−0.0061 (10)
C6	0.0423 (8)	0.0512 (8)	0.0546 (10)	−0.0051 (7)	−0.0020 (8)	−0.0051 (8)
C7	0.0439 (8)	0.0510 (8)	0.0507 (9)	−0.0073 (7)	0.0006 (8)	−0.0013 (8)
C8	0.0507 (8)	0.0514 (8)	0.0457 (10)	−0.0020 (7)	0.0026 (7)	0.0047 (7)
C9	0.0464 (8)	0.0517 (9)	0.0432 (10)	−0.0046 (7)	−0.0041 (8)	0.0013 (7)
C10	0.0423 (8)	0.0544 (9)	0.0462 (10)	0.0012 (7)	−0.0057 (7)	−0.0015 (7)
C11	0.0543 (10)	0.0713 (11)	0.0494 (10)	0.0017 (8)	−0.0034 (9)	0.0027 (9)
C12	0.0578 (11)	0.0870 (14)	0.0462 (11)	0.0111 (9)	0.0013 (9)	−0.0042 (9)
C13	0.0558 (10)	0.0756 (12)	0.0544 (12)	0.0154 (8)	−0.0081 (9)	−0.0164 (9)
C14	0.0462 (9)	0.0547 (10)	0.0551 (11)	0.0043 (7)	−0.0095 (8)	−0.0082 (8)
C15	0.0448 (9)	0.0556 (10)	0.0504 (10)	0.0016 (7)	−0.0024 (8)	−0.0039 (8)
C16	0.0820 (14)	0.0548 (10)	0.0791 (16)	−0.0039 (9)	−0.0051 (12)	0.0030 (10)
N	0.0935 (12)	0.0494 (7)	0.0455 (9)	0.0024 (7)	0.0110 (8)	−0.0002 (6)
O1	0.1073 (10)	0.0510 (6)	0.0485 (8)	0.0060 (7)	0.0055 (7)	0.0026 (5)
O2	0.0790 (9)	0.0518 (7)	0.0805 (10)	0.0030 (6)	0.0026 (8)	−0.0072 (7)

### Geometric parameters (Å, º)

**Table:**

C1—C2	1.380 (3)	C9—C10	1.469 (2)
C1—C6	1.389 (3)	C10—C11	1.383 (2)
C1—H1	0.9300	C10—C15	1.400 (2)
C2—C3	1.376 (3)	C11—C12	1.384 (3)
C2—H2	0.9300	C11—H11	0.9300
C3—C4	1.369 (3)	C12—C13	1.380 (3)
C3—H3	0.9300	C12—H12	0.9300
C4—C5	1.394 (3)	C13—C14	1.381 (3)
C4—H4	0.9300	C13—H13	0.9300
C5—C6	1.385 (3)	C14—O2	1.370 (2)
C5—H5	0.9300	C14—C15	1.384 (2)
C6—C7	1.480 (2)	C15—H15	0.9300
C7—N	1.311 (2)	C16—O2	1.413 (3)
C7—C8	1.398 (2)	C16—H16A	0.9600
C8—C9	1.363 (2)	C16—H16B	0.9600
C8—H8	0.9300	C16—H16C	0.9600
C9—O1	1.331 (2)	N—O1	1.4165 (19)
			
C2—C1—C6	120.42 (19)	C11—C10—C15	120.20 (16)
C2—C1—H1	119.8	C11—C10—C9	120.10 (15)
C6—C1—H1	119.8	C15—C10—C9	119.70 (16)
C3—C2—C1	120.3 (2)	C10—C11—C12	119.55 (18)
C3—C2—H2	119.8	C10—C11—H11	120.2
C1—C2—H2	119.8	C12—C11—H11	120.2
C4—C3—C2	119.96 (19)	C13—C12—C11	120.50 (19)
C4—C3—H3	120.0	C13—C12—H12	119.7
C2—C3—H3	120.0	C11—C12—H12	119.7
C3—C4—C5	120.2 (2)	C12—C13—C14	120.10 (17)
C3—C4—H4	119.9	C12—C13—H13	119.9
C5—C4—H4	119.9	C14—C13—H13	119.9
C6—C5—C4	120.08 (19)	O2—C14—C13	115.50 (16)
C6—C5—H5	120.0	O2—C14—C15	124.32 (17)
C4—C5—H5	120.0	C13—C14—C15	120.18 (17)
C5—C6—C1	118.94 (16)	C14—C15—C10	119.45 (17)
C5—C6—C7	120.71 (16)	C14—C15—H15	120.3
C1—C6—C7	120.35 (17)	C10—C15—H15	120.3
N—C7—C8	111.07 (14)	O2—C16—H16A	109.5
N—C7—C6	119.20 (16)	O2—C16—H16B	109.5
C8—C7—C6	129.71 (16)	H16A—C16—H16B	109.5
C9—C8—C7	105.12 (15)	O2—C16—H16C	109.5
C9—C8—H8	127.4	H16A—C16—H16C	109.5
C7—C8—H8	127.4	H16B—C16—H16C	109.5
O1—C9—C8	109.67 (16)	C7—N—O1	105.89 (14)
O1—C9—C10	117.72 (15)	C9—O1—N	108.24 (14)
C8—C9—C10	132.60 (16)	C14—O2—C16	118.23 (14)
			
C6—C1—C2—C3	1.4 (3)	C8—C9—C10—C15	162.56 (17)
C1—C2—C3—C4	−0.4 (3)	C15—C10—C11—C12	−0.1 (2)
C2—C3—C4—C5	−0.8 (3)	C9—C10—C11—C12	178.65 (16)
C3—C4—C5—C6	1.1 (3)	C10—C11—C12—C13	0.0 (3)
C4—C5—C6—C1	−0.1 (3)	C11—C12—C13—C14	−0.7 (3)
C4—C5—C6—C7	179.55 (18)	C12—C13—C14—O2	−177.92 (15)
C2—C1—C6—C5	−1.1 (3)	C12—C13—C14—C15	1.6 (2)
C2—C1—C6—C7	179.22 (17)	O2—C14—C15—C10	177.77 (14)
C5—C6—C7—N	14.4 (2)	C13—C14—C15—C10	−1.7 (2)
C1—C6—C7—N	−165.93 (17)	C11—C10—C15—C14	0.9 (2)
C5—C6—C7—C8	−164.38 (18)	C9—C10—C15—C14	−177.80 (14)
C1—C6—C7—C8	15.3 (3)	C8—C7—N—O1	−0.2 (2)
N—C7—C8—C9	−0.4 (2)	C6—C7—N—O1	−179.16 (14)
C6—C7—C8—C9	178.48 (16)	C8—C9—O1—N	−0.93 (19)
C7—C8—C9—O1	0.81 (18)	C10—C9—O1—N	178.15 (13)
C7—C8—C9—C10	−178.09 (17)	C7—N—O1—C9	0.7 (2)
O1—C9—C10—C11	165.00 (15)	C13—C14—O2—C16	−179.83 (16)
C8—C9—C10—C11	−16.2 (3)	C15—C14—O2—C16	0.7 (2)
O1—C9—C10—C15	−16.3 (2)		

### Hydrogen-bond geometry (Å, º)

*Cg* is the centroid of the C1–C6 ring.

**Table:**

*D*—H···*A*	*D*—H	H···*A*	*D*···*A*	*D*—H···*A*
C1—H1···*Cg*^i^	0.93	2.96	3.732 (2)	141
C4—H4···*Cg*^ii^	0.93	3.06	3.768 (3)	134

Symmetry codes: (i) −*x*+1, −*y*+2, *z*−1/2; (ii) −*x*, −*y*+2, *z*+1/2.
